# A new bilaterally injured trilobite presents insight into attack patterns of Cambrian predators

**DOI:** 10.7717/peerj.14185

**Published:** 2022-10-10

**Authors:** Ruiwen Zong, Russell D.C. Bicknell

**Affiliations:** 1 State Key Laboratory of Biogeology and Environmental Geology, China University of Geosciences, Wuhan, China; 2 Palaeoscience Research Centre, School of Environmental and Rural Science, University of New England, New South Wales, Australia

**Keywords:** Predation, Regeneration, *Redlichia*, Cambrian, South China

## Abstract

Durophagous predation in the Cambrian is typically recorded as malformed shells and trilobites, with rarer evidence in the form of coprolites and shelly gut contents. Reporting novel evidence for shell-crushing further expands the understanding of where and when in the Cambrian durophagy was present. To expand the current documentation and present new records of malformed trilobites from the Cambrian of China, we present an injured *Redlichia* (*Pteroredlichia*) *chinensis* from the lower Cambrian Balang Formation, western Hunan, South China. The specimen has two distinct injuries along the thorax. The injuries show different degrees of regeneration, suggesting that the specimen was attacked twice. We propose that the individual may have been targeted more readily for the second attack. This predatory approach would have been highly energy efficient, maximizing net energy gain during the attack.

## Introduction

Biomineralized trilobite exoskeleton was constructed from two layers of low magnesium calcite (Wilmot & Fallick, 1989; Hughes, 2007). Due to this construction, trilobites had a markedly durable dorsal exoskeleton when compared to most other Paleozoic euarthropods (Hughes, 2007). Although this cuticular construction protected the soft-bodied sections, these exoskeletons were still susceptible to damage from shell crushing (durophagous) predators and boring organisms especially post-molting, during the soft- and paper-shell stages (*e.g.*, Owen, 1985; Babcock, 1993; Pratt, 1998; Zamora et al., 2011; Fatka, Budil & Grigar, 2015; Pates et al., 2017; Bicknell & Paterson, 2018; Bicknell & Pates, 2020; De Baets et al., 2021). This is evidence by the recorded in malformed and injured specimens (Owen, 1985). These injuries provide insight into Paleozoic predation strategies, predator–prey interactions, evolution of trilobite morphology and behavior, and Paleozoic foodwebs (Babcock & Robison, 1989; Babcock, 1993; Babcock, 2003; Babcock, 2007; Nedin, 1999; Brett & Walker, 2002; Brett, 2003; Bicknell & Paterson, 2018; Bicknell et al., 2022a).

While there have been rare instances of accidental injuries (Rudkin, 1985), the majority injured Cambrian and Ordovician trilobites are attributed to failed predation (Owen, 1985; Babcock, 1993; Bicknell & Paterson, 2018; Bicknell et al., 2021; Bicknell et al., 2022a). Within this fossil record, most injured specimens show unilaterally expressed injuries (Babcock, 1993; Bicknell & Paterson, 2018 and their references), with rarer evidence of multiple injuries across the exoskeleton (*e.g.*, Šnajdr, 1979; Conway Morris & Jenkins, 1985; Ou et al., 2009; Pates & Bicknell, 2019; Bicknell & Holland, 2020; Bicknell & Pates, 2020; Bicknell et al., 2021; Bicknell et al., 2022a). Determining whether specimens with multiple injuries record one or multiple failed attacks is complex. However, the extent of exoskeletal regeneration can be considered a proxy for understanding the timing of attacks and subsequent recovery (*e.g.*, Cheng et al., 2019; Zong, 2021a). To expand on this line of enquiry, we describe a malformed redlichiid trilobite from the Cambrian-aged Balang Formation (western Hunan, South China). This specimen shows a bilaterally expressed malformation and is used to explore patterns of trilobite regeneration, presenting new insight into early Cambrian predation strategies.

## Materials and Methods

**Figure 1:**
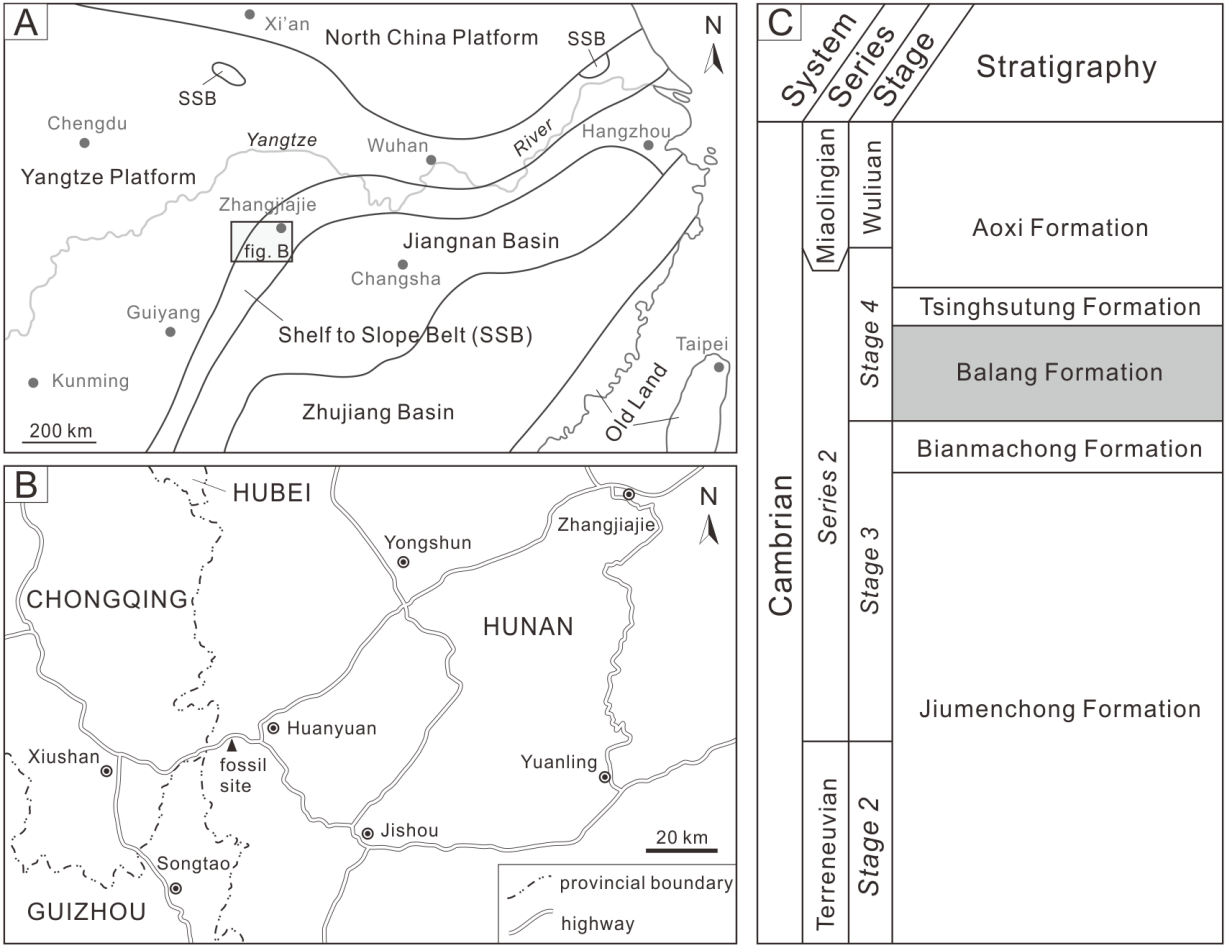
Geological background map of study area and location of the fossil site. (A) Cambrian sedimentary facies zones of South China (modified from Zhao et al., 1993). (B) Map of fossil site at Huayuan County, Xiangxi Autonomous Prefecture, Hunan Province. (C) Stratigraphic series showing relative position and age of the Balang Formation (modified from Zhu et al., 2021).

The examined specimen was collected from the Balang Formation, Huayuan County, Xiangxi Autonomous Prefecture, Hunan Province (Fig. 1). The Balang Formation is widely distributed in eastern Guizhou and northwestern Hunan, and is comprised of fine calcareous clastic rocks with limited limestone interbeds or lenses. The formation was therefore likely deposited in a shelf to slope environment (Fig. 1; Yin, 1996; Liang et al., 2017). The formation is located within the *Arthricocephalus chauveaui-Changaspis elongata* zone, which corresponds to the Cambrian *Series 2, Stage 4* (Peng, 2009; Qin et al., 2010). The Balang Formation has yielded a diverse, exceptionally preserved fauna including radiodonts, trilobitomorphs, bivalved arthropods, worms, chancelloriids, cnidarians, echinoderms, and algae (Peng et al., 2005; Liu & Lei, 2013). Trilobites from this formation consist of primarily oryctocephalids, redlichiids, and ptychopariids that are arrayed across ten genera (Peng et al., 2018; Chen, 2019). The Balang redlichiids consist of four species (including one subspecies) within a *Redlichia* subgenus (Liang et al., 2017; Chen & Zhao, 2018), and the dominate taxon is *Redlichia* (*Pteroredlichia*) *chinensis* (Walcott, 1905). The dark gray calcareous shale of the Balang Formation in western Hunan has yielded a large number of well-preserved *R*. (*Pteroredlichia*) *chinensis* (Zong, 2021b). The malformed individual was assigned to this species and represents an internal mold of *R*. (*Pteroredlichia*) *chinensis*. The examined specimen was coated with magnesium oxide for photography. All photographs were taken with a Nikon D5100 camera using a Micro-Nikkor 55 mm F3.5 lens. Specimen measurements were made with ImageJ software (Schneider, Rasband & Eliceiri, 2012). The specimen is housed in the State Key Laboratory of Biogeology and Environmental Geology, China University of Geosciences (Wuhan) (BGEG).

## Results

**Figure 2:**
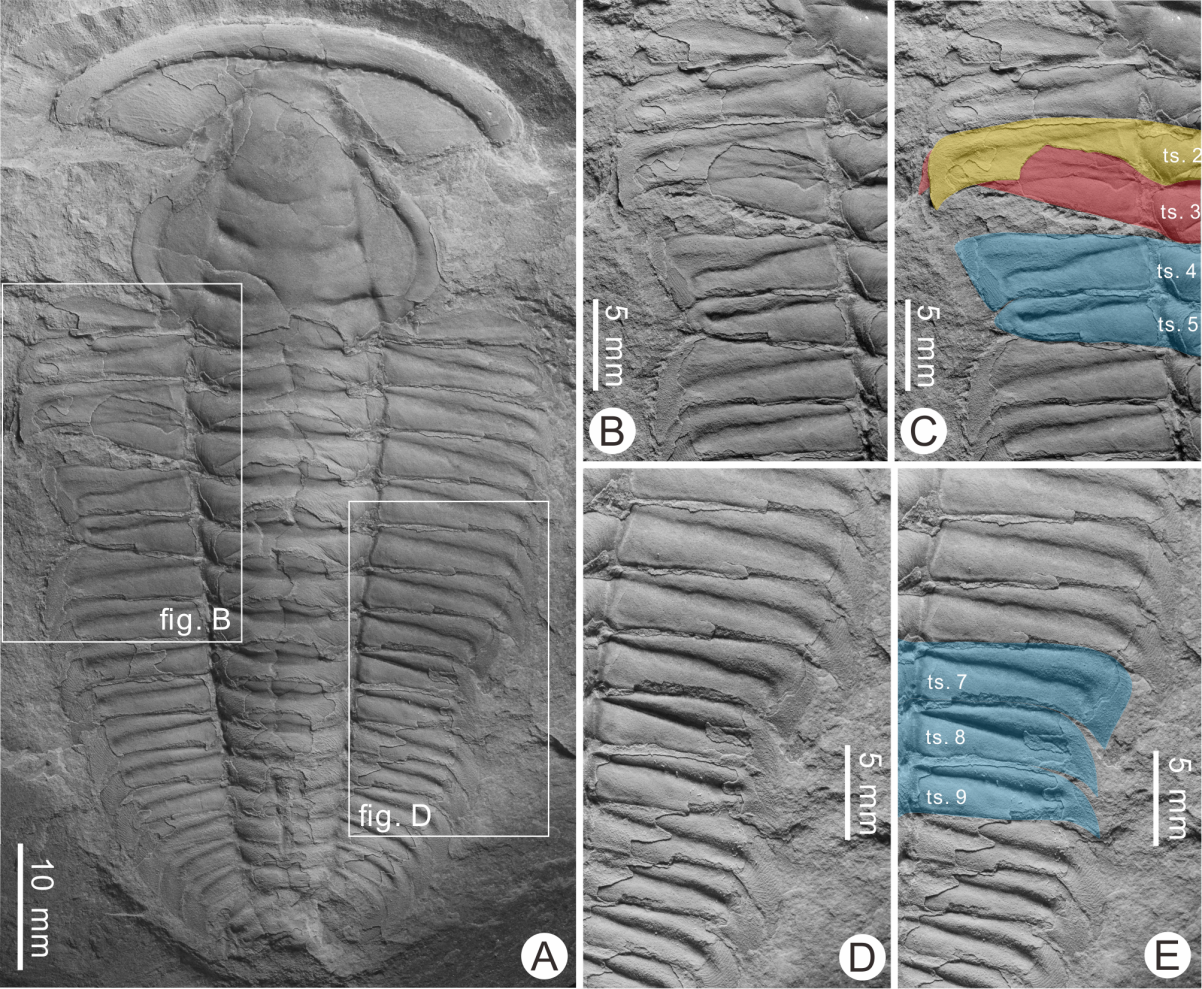
Injured *Redlichia* (*Pteroredlichia*) *chinensis* (Walcott, 1905) from the Balang Formation (Cambrian *Series 2*, *Stage 4*), Hunan, South China. (A) Complete specimen (BGEG-HXB-02) showing two injuries. (B, C) Close-up of the injury on the left pleural lobe. (B) V-shaped indentation. (C) Same as B showing overlap of the second (yellow) and third (red) pleural segments and injury (blue). (D, E) Close-up of injury on right pleural lobe. (D) U-shaped indentation. (E) Same as D showing injury (blue). Abbreviation: ts., thoracic segment.

The described specimen (BGEG-HXB-02) is an articulated exoskeleton lacking free cheeks and is therefore likely an exuvia (Daley & Drage, 2016; Drage, 2019). Two malformations along the thorax are noted (Fig. 2A). The more anterior malformation is an asymmetrical V-shaped indentation along the fourth and fifth pleural segments of the left pleural lobe showing limited cicatrization (Fig. 2B). The distal section of the fourth pleural segment is lacking a pleural spine, truncated by ∼1.8 mm. The pleural furrow of this segment is slightly S-shaped. The fifth thoracic segment is truncated by ∼2.9 mm and reduced abaxially. Distal section of this segment is rounded and reduced by ∼50% relative to the undamaged sixth pleural segment. The pleural furrow of this segment is also slightly distorted. Additionally, the third segment on the left side was rotated anteriorly and positioned under the second pleural segment. As this rotated segment lacks shortening or distortion (Fig. 2C), this reflects either taphonomic alteration or displacement during molting (Zong, 2021a).

The second malformation is a U-shaped indentation located across the seventh to ninth pleural segments on the right pleural lobe (Figs. 2D and 2E). The seventh and eight segments are truncated by ∼1.8 mm and ∼2.6 mm, respectively. The ninth segment is truncated by 2.5 mm, and the distal portion is narrower when compared to the ninth segment on the left pleural lobe. All malformed segments have reduced pleural spines that are ∼50% smaller when compared to undamaged segments, indicating pleural spine regeneration (Pates et al., 2017).

## Discussion

Several factors likely produced malformations in trilobites. These include failed predation or molting resulting in injuries, developmental malformations producing teratologies, and pathological infections (*e.g.*, Šnajdr, 1978; Rudkin, 1985; Owen, 1985; Babcock, 1993; Babcock, 2003; Owen & Tilsley, 1996; Chen, 2011; Fatka, Budil & Grigar, 2015; Bicknell & Pates, 2020; Bicknell & Smith, 2021; De Baets et al., 2021; Zong, 2021b). Morphologically comparable abnormalities are observed in modern arthropods (Juanes & Smith, 1995; Brandt, 2002; Pandourski & Evtimova, 2005; Pandourski & Evtimova, 2009; Hopkins & Das, 2015; Bicknell, Pates & Botton, 2018; Bicknell et al., 2022b; Das et al., 2021; De Baets et al., 2021), supporting the hypotheses that trilobites experienced similar malformations as extant species. Abnormal trilobite and horseshoe crab specimens with U-, V- or W-shaped breakages, reduced segments, and evidence of cicatrization or regeneration are considered indicative of non-lethal predation (Šnajdr, 1978; Babcock, 1993; Nedin, 1999; Zhu et al., 2007; Bicknell & Paterson, 2018; Bicknell, Pates & Botton, 2018; Bicknell et al., 2022a; Pates & Bicknell, 2019; Bicknell & Pates, 2020; Zong, 2021b). Two indentations documented here are V- and U-shaped with cicatrization on the left injury and pleural spine regeneration on the right injury. Given this, we confidently assign these indentations to failed predation within the Balang Formation. More importantly, these injuries show distinctly different stages of recovery (Fig. 2).

Trilobites are thought to have recovered from injury in an antero-posterior manner, such that anterior segments show markedly more recovery than posterior sections (*e.g.*, Šnajdr, 1981; Conway Morris & Jenkins, 1985; Babcock, 1993; McNamara & Tuura, 2011). This pattern is common to modern arthropods and annelids and is controlled by segmentation polarity genes (McNamara & Tuura, 2011). In BGEG-HXB-02, the anterior injury shows cicatrization, but no evidence for segment regeneration. This presents an apparent conundrum. If we assume the injuries were incurred at the same time, we directly contradict fundamental theories on arthropod development (McNamara & Tuura, 2011). The most parsimonious explanation for the observed pattern is that the posterior injury was incurred first. This injury was able to regenerate before the more anterior injury was incurred.

The presence of two injuries from two distinct attacks demonstrates that Cambrian trilobites could have experienced multiple attacks during their life cycle. This has important implications for Cambrian predator–prey systems, especially with comparison to modern systems. Extant predators in both terrestrial and marine ecosystems will target weaker prey within a population as these individuals require less energy in predation and likely represent a more successful attack (Temple, 1987; Mesa et al., 1994; Hethcote et al., 2004; Peharda & Morton, 2006; Genovart et al., 2010). It seems that predation targeting more vulnerable individuals had therefore arisen in the Cambrian and may have allowed the first durophages to maximizing net energy gain (*i.e.,* energy derived from prey after accounting for energy lost during predation; Forsman, 1996; Gosselin & Chia, 1996) during predation. Finally, the rarity of trilobite specimens with multiple distinct injuries likely reflects an increased rate of successful predation, and a higher rate of mortality in previously injured individuals.

## Additional Information and Declarations

### Data Availability

The following information was supplied regarding data availability: The raw data are available in Figures 1 and 2. The specimen is stored in the State Key Laboratory of Biogeology and Environmental Geology, China University of Geosciences, Wuhan, China: BGEG-HXB-02.

## References

[ref-1] Babcock LE (1993). Trilobite malformations and the fossil record of behavioral asymmetry. Journal of Paleontology.

[ref-2] Babcock LE, Kelley PH, Kowalewski M, Hansen TA (2003). Trilobites in Paleozoic predatorprey systems, and their role in reorganization of early Paleozoic ecosystems. Predator–prey interactions in the fossil record.

[ref-3] Babcock LE, Mikulic DG, Landing E, Kluessendorf J (2007). Role of malformations in elucidating trilobite paleobiology: a historical synthesis. Fabulous fossils—300 years of worldwide research on trilobites.

[ref-4] Babcock LE, Robison RA (1989). Preferences of Palaeozoic predators. Nature.

[ref-5] Bicknell RDC, Holland B (2020). Injured trilobites within a collection of dinosaurs: using the royal tyrrell museum of palaeontology to document Cambrian predation. Palaeontologia Electronica.

[ref-6] Bicknell RDC, Holmes JD, Pates S, García-Bellido DC, Paterson JR (2022a). Cambrian carnage: trilobite predator–prey interactions in the Emu Bay Shale of South Australia. Palaeogeography, Palaeoclimatology, Palaeoecology.

[ref-7] Bicknell RDC, Paterson JR (2018). Reappraising the early evidence of durophagy and drilling predation in the fossil record: implications for escalation and the Cambrian Explosion. Biological Reviews.

[ref-8] Bicknell RDC, Pates S (2020). Exploring abnormal Cambrian-aged trilobites in the Smithsonian collection. PeerJ.

[ref-9] Bicknell RDC, Pates S, Botton ML (2018). Abnormal xiphosurids, with possible application to Cambrian trilobites. Palaeontologia Electronica.

[ref-10] Bicknell RDC, Pates S, Kaiser D, Zakrzewski S, Botton ML, Tanacredi JT, Botton ML, Shin PKS, Iwasaki Y, Cheung SG, Kwan KY, Mattei JH (2022b). Applying records of extant and extinct horseshoe crab abnormalities to xiphosurid conservation.

[ref-11] Bicknell RDC, Smith PM (2021). Teratological trilobites from the Silurian (Wenlock and Ludlow) of Australia. The Science of Nature.

[ref-12] Bicknell RDC, Smith PM, Bruthansová J, Holland B (2021). Malformed trilobites from the Ordovician and Devonian. Paläontologische Zeitschrift.

[ref-13] Brandt DS (2002). Ecydsial efficiency and evolutionary efficacy among marine arthropods: Implications for trilobite survivorship. Alcheringa.

[ref-14] Brett CE, Kelley PH, Kowalewski M, Hansen TA (2003). Durophagous predation in Paleozoic marine benthic assemblages. Predator–prey interactions in the fossil record.

[ref-15] Brett CE, Walker SE (2002). Predators and predation in Paleozoic marine environments. Paleontological Society Papers.

[ref-16] Chen GY (2011). The Silurian trilobite *Coronocephalus gaoluoensis* Wu from western Hunan: its features, exuviation and abnormalities. PhD Thesis.

[ref-17] Chen ZP (2019). The trilobite biostratigraphy of the Cambrian Series 2, Stage 4 in Jianhe County, Guizhou Province. Master Thesis.

[ref-18] Chen ZP, Zhao YL (2018). Trilobite *Redlichia* from the Cambrian Tsinghsutung Formation (*Series 2, Stage 4*) of Eastern Guizhou, South China. Journal of Guizhou University (Natural Sciences).

[ref-19] Cheng MR, Han J, Ou Q, Zhang ZF, Guo J, Zhang XL (2019). Injured trilobite *Eoredichia intermedia* from the Early Cambrian Chengjiang biota. Acta Palaeontologica Sinica.

[ref-20] Conway Morris S, Jenkins RJF (1985). Healed injuries in early Cambrian trilobites from South Australia. Alcheringa.

[ref-21] Daley AC, Drage HB (2016). The fossil record of ecdysis, and trends in the moulting behaviour of trilobites. Arthropod Structure & Development.

[ref-22] Das S, Tripathy B, Subramanian KA, Bicknell RDC (2021). On abnormal *Carcinoscorpius rotundicauda* (Latreille, 1802)(Chelicerata: Xiphosurida) from the Indian Sundarbans and possible conservation directions. Arthropoda Selecta.

[ref-23] De Baets K, Budil P, Fatka O, Geyer G, De Baets K, Huntley JW (2021). Trilobites as hosts for parasites: from paleopathologies to etiologies. The evolution and fossil record of parasitism: coevolution and paleoparasitological techniques.

[ref-24] Drage HB (2019). Quantifying intra- and interspecific variability in trilobite moulting behaviour across the Palaeozoic. Palaeontologia Electronica.

[ref-25] Fatka O, Budil P, Grigar L (2015). A unique case of healed injury in a Cambrian trilobite. Annales de Paleontologie.

[ref-26] Forsman A (1996). Body size and net energy gain in gape-limited predators: a model. Journal of Herpetology.

[ref-27] Genovart M, Negre N, Tavecchia G, Bistuer A, Parpal L, Oro D (2010). The young, the weak and the sick: evidence of natural selection by predation. PLOS ONE.

[ref-28] Gosselin LA, Chia F-S (1996). Prey selection by inexperienced predators: do early juvenile snails maximize net energy gains on their first attack?. Journal of Experimental Marine Biology and Ecology.

[ref-29] Hethcote HW, Wang W, Han L, Ma Z (2004). A predator–prey model with infected prey. Theoretical Population Biology.

[ref-30] Hopkins PM, Das S, Chang ES, Thiel M (2015). Regeneration in crustaceans. The natural history of the Crustacea.

[ref-31] Hughes NC (2007). The evolution of trilobite body patterning. Annual Review of Earth and Planetary Sciences.

[ref-32] Juanes F, Smith LD (1995). The ecological consequences of limb damage and loss in decapod crustaceans: a review and prospectus. Journal of Experimental Marine Biology and Ecology.

[ref-33] Liang BY, Peng J, Wen RQ, Liu S (2017). Ontogeny of the trilobite *Redlichia* (*Pteroredlichia*) *chinensis* (Walcott, 1905) from the Cambrian Balang Formation. Acta Palaeontologica Sinica.

[ref-34] Liu Q, Lei QP (2013). Discovery of an exceptionally preserved fossil assemblage in the Balang Formation (Cambrian *Series 2*, Stage 4) in Hunan, China. Alcheringa.

[ref-35] McNamara KJ, Tuura ME (2011). Evidence for segment polarity during regeneration in the Devonian asteropygine trilobite *Greenops widderensis*. Journal of Paleontology.

[ref-36] Mesa MG, Poe TP, Gadomski DM, Petersen JH (1994). Are all prey created equal? A review and synthesis of differential predation on prey in substandard condition. Journal of Fish Biology.

[ref-37] Nedin C (1999). Anomalocaris predation on nonmineralized and mineralized trilobites. Geology.

[ref-38] Ou Q, Shu DG, Han J, Zhang XL, Zhang ZF, Liu JN (2009). A juvenile redlichiid trilobite caught on the move: evidence from the Cambrian (*Series 2*) Chengjiang Lagerstätte, southwestern China. Palaios.

[ref-39] Owen AW (1985). Trilobite abnormalities. Transactions of the Royal Society of Edinburgh: Earth Sciences.

[ref-40] Owen AW, Tilsley JW (1996). An abnormal pygidium of the trilobite *Brachymetopus ornatus* Woodward from the Lower Carboniferous of Derbyshire. Geological Journal.

[ref-41] Pandourski I, Evtimova V (2005). Teratological morphology of copepods (Crustacea) from Iceland. Acta Zoologica Bulgarica.

[ref-42] Pandourski I, Evtimova V (2009). Morphological variability and teratology of lower crustaceans (Copepoda and Branchiopoda) from circumpolar regions. Acta Zoologica Bulgarica.

[ref-43] Pates S, Bicknell RDC (2019). Elongated thoracic spines as potential predatory deterrents in olenelline trilobites from the lower Cambrian of Nevada. Palaeogeography, Palaeoclimatology, Palaeoecology.

[ref-44] Pates S, Bicknell RDC, Daley AC, Zamora S (2017). Quantitative analysis of repaired and unrepaired damage to trilobites from the Cambrian (*Stage 4*, Drumian) Iberian Chains, NE Spain. Palaios.

[ref-45] Peharda M, Morton B (2006). Experimental prey species preferences of *Hexaplex trunculus* (Gastropoda: Muricidae) and predator–prey interactions with the Black mussel *Mytilus galloprovincialis* (Bivalvia: Mytilidae). Marine Biology.

[ref-46] Peng J (2009). The Qiandongian (Cambrian) Balang Fauna from eastern Guizhou, South China. PhD Thesis.

[ref-47] Peng SC, Babcock LE, Zhu XJ, Dai T (2018). A new oryctocephalid trilobite from the Balang Formation (Cambrian *Stage 4*) of northwestern Hunan, South China, with remarks on the classification of oryctocephalids. Palaeoworld.

[ref-48] Peng J, Zhao YL, Wu YS, Yuan JL, Tai TS (2005). The Balang Fauna—a new early Cambrian fauna from Kaili City, Guizhou Province. Chinese Science Bulletin.

[ref-49] Pratt BR (1998). Probable predation on Upper Cambrian trilobites and its relevance for the extinction of soft-bodied Burgess Shale-type animals. Lethaia.

[ref-50] Qin Q, Peng J, Fu XP, Da Y (2010). Restudy of *Changaspis* (Lee), 1961 from Qiandongian (Early Cambrian) Balang Formation near eastern Guizhou, south China. Acta Plaeontological Sinica.

[ref-51] Rudkin DM (1985). Exoskeletal abnormalities in four trilobites. Canadian Journal of Earth Sciences.

[ref-52] Schneider CA, Rasband WS, Eliceiri KW (2012). NIH Image to ImageJ: 25 years of image analysis. Nature Methods.

[ref-53] Šnajdr M (1978). Anomalous carapaces of Bohemian paradoxid trilobites. Sborník geologických věd, Paleontologie.

[ref-54] Šnajdr M (1979). Two trinucleid trilobites with repair of traumatic injury. Věstník Ústředního ústavu geologického.

[ref-55] Šnajdr M (1981). Bohemian Proetidae with malformed exoskeletons (Trilobita). Sborník Geologických Věd Paleontologie.

[ref-56] Temple SA (1987). Do predators always capture substandard individuals disproportionately from prey populations?. Ecology.

[ref-57] Walcott CD (1905). Cambrian faunas of China. Proceedings of the United States National Museum.

[ref-58] Wilmot NV, Fallick AE (1989). Original mineralogy of trilobite exoskeletons. Palaeontology.

[ref-59] Yin GZ (1996). Division and correlation of Cambrian in Guizhou. Guizhou Geology.

[ref-60] Zamora S, Mayoral E, Esteve J, Gámez-Vintaned JA, Santos A (2011). Exoskeletal abnormalities in paradoxidid trilobites from the Cambrian of Spain, and a new type of bite trace. Bulletin of Geosciences.

[ref-61] Zhao YL, Yuan JL, Zhang ZH, Mao JR, Huang YZ, Gong XY, Wang K (1993). Preliminary study of the Kaili Formation and its synchronous strata in the transitional belt of South China. Journal of Stratigraphy.

[ref-62] Zhu XJ, Peng SC, Du SX, Hu YS (2007). Ontogeny and malformation of *Tamdaspis jingxiensis* sp. nov. (trilobite, Cambrian) from Jingxi, Guangxi, China. Acta Palaeontologica Sinica.

[ref-63] Zhu MY, Sun ZX, Yang AH, Yuan JL, Li GX, Zhou ZQ, Zhang JM (2021). Lithostratigraphic subdivision and correlation of the Cambrian in China. Journal of Stratigraphy.

[ref-64] Zong RW (2021a). Injuries and molting interference in a trilobite from the Cambrian (Furongian) of South China. PeerJ.

[ref-65] Zong RW (2021b). Abnormalities in early Paleozoic trilobites from central and eastern China. Palaeoworld.

